# The internal thoracic artery skeletonization study: A paired, within-patient comparison [NCT00265499]

**DOI:** 10.1186/1745-6215-7-1

**Published:** 2006-01-05

**Authors:** Munir Boodhwani, Howard J Nathan, B Khanh Lam, Fraser D Rubens

**Affiliations:** 1 Division of Cardiac Surgery, University of Ottawa Heart Institute, Ottawa, Canada; 2 Division of Cardiac Anesthesia, University of Ottawa Heart Institute, Ottawa, Canada

## Abstract

**Background:**

Traditional harvesting of the internal thoracic artery (ITA) for use as a conduit in coronary bypass surgery involves the dissection of a rim of tissue surrounding the artery on either side. Recent studies, primarily observational, have suggested that skeletonization of the ITA can improve conduit flow, increase length, and reduce the risk of deep sternal infection in high risk patients. Furthermore, skeletonization of the ITA can potentially preserve intercostal nerves and reduce post-operative pain and dysesthesias associated with ITA harvesting. In order to assess the effects of ITA skeletonization, we report a prospective, randomized, within-patient study design that shares many features of a cross-over study.

**Methods:**

Patients undergoing bilateral internal thoracic artery harvest will be randomized to having one side skeletonized and the other harvested in a non-skeletonized manner. Outcome measures include ITA flow and length measured intra-operatively, post-operative pain and dysesthesia, evaluated at discharge, four weeks, and three months post-operatively, and sternal perfusion assessed using single photon emission computed tomography. Harvest times as well as safety endpoints of ITA injury will be recorded.

**Discussion:**

This study design, using within-patient comparisons and paired analyses, minimizes the variability of the outcome measures, which is seldom possible in the evaluation of surgical techniques, with minimal chance of carryover effects that can hamper the interpretation of traditional cross-over studies. This study will provide a valid evaluation of clinically relevant effects of internal thoracic artery skeletonization in improving outcomes following coronary artery bypass surgery.

## Introduction

Coronary artery bypass surgery (CABG) remains the gold standard for the treatment of multi-vessel and left main coronary disease[1]. The use of the internal thoracic arteries (ITAs) as bypass conduits has been associated with improved long-term outcome following CABG[2,3]. Despite these long-term benefits, there are certain characteristics of the ITAs that limit their use in particular settings. The internal thoracic arteries can be prone to vasospasm and hypoperfusion in the early post-operative period, particularly in the presence of vasoactive medications[4,5]. Harvest of the ITAs is associated with impairment in sternal perfusion[6] and increased deep sternal wound infections[7], particularly in diabetic patients. The length of usable conduit available is often limited and ITA harvest has been associated with increased and persistent post-operative pain [8-10].

The traditional ITA harvesting technique involves the dissection of a rim of tissue (1 – 2 cm) around the ITA. Skeletonization of the ITA has been proposed as a solution to many of the problems associated with ITA harvesting. The skeletonization procedure, first described by Keeley et al in 1987[11], involves the harvest of only the ITA without any surrounding tissue. This technique requires meticulous dissection and carries the theoretical risk of increased arterial injury. However, studies to date have not demonstrated any differences in microscopic injury or vascular function when compared to non-skeletonized ITAs[12,13]. Numerous benefits of skeletonized ITA harvest have been proposed including increased flow[14] and length[12], as well as a decreased sternal infection rates[15], and reduced pain[16]. However, these assertions are supported primarily by non-randomized, observational studies. It is not surprising, therefore, that there is significant variation among surgeons as to the harvesting technique employed.

In order to evaluate the differences in skeletonized versus non-skeletonized ITA harvest, we propose the use of a paired study design to evaluate within-patient differences in ITA flow, length, sternal perfusion, and post-operative pain and dysesthesias.

## Methods

### Patient population

This study has been approved by the Institutional Review Board of the University of Ottawa Heart Institute. All patients undergoing isolated, non-emergent coronary artery bypass surgery using bilateral internal thoracic artery harvest by two surgeons will be screened for eligibility for this study. Exclusion criteria include known subclavian artery stenosis, non-fluency in English or French, and inability to complete follow-up visits. Of note, insulin-dependent diabetes, systemic steroid use, and morbid obesity remain relative contra-indications for bilateral non-skeletonized internal thoracic artery use at our institution.

### Study design, randomization, and blinding

This is a prospective, randomized study in which each patient receives both study interventions i.e. a skeletonized ITA and a non-skeletonized ITA. Patients are randomly allocated to receive a skeletonized ITA first followed by a non-skeletonized harvest or vice versa i.e. the order of treatments is randomly assigned. In addition, the randomization scheme is stratified by the side to which the intervention is applied in order to ensure equivalent numbers of left and right sided ITAs that are skeletonized. Randomization will be performed intra-operatively by opening a sealed, opaque envelope at the time of sternotomy which contains computer-generated random sequences assigned to each enrolled patient.

Patients will remain unaware of the treatment assignment throughout the course of the study. Assessment of the outcome measures will be performed by individuals blinded to the treatment assignment whenever possible. Because of the nature of certain endpoints, requiring visual feedback, evaluation of ITA length and flow will be performed intra-operatively by individuals performing the ITA harvest who will be aware of the treatment assignment.

### Outcome measures

**Figure 1:**
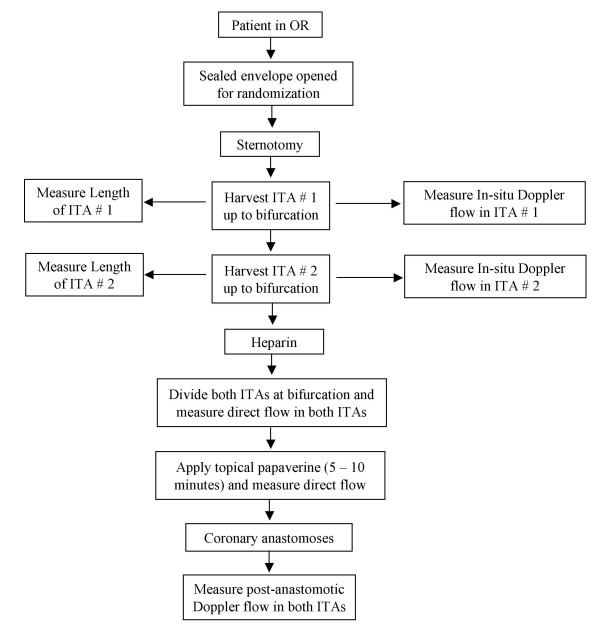
The intra-operative protocol involves measurement of ITA length (in-situ) as well as intra-operative flow measurements on four occasions.

The conduct of the intra-operative procedures as well as the intra-operative outcomes is displayed in figure 1.

### Conduit length

The length of the harvested ITA will be measured in-situ (i.e. with the proximal and distal ends attached) from the first rib to ITA bifurcation in a standardized fashion. After the conduit has been fully dissected along its entire length and prior to division of its distal end, the midportion will be encircled by a length of sterile silk suture. Uniform tension will be applied to the two ends of the suture. A separate silk suture will then be used follow the course of the ITA to measure its length from the first rib to the ITA bifurcation. Separate sutures will be used for the left and right sides and labeled in order to avoid misclassification.

### Conduit flow

ITA flow will be measured on four separate occasions. The first time point will be immediately following each individual ITA harvest with the vessel in-situ, using a Doppler flow meter (Transonics Inc., Binghamton, NY, USA). The flow probe will be placed at a point approximately halfway between the first rib and the ITA bifurcation. The size of the probe will be chosen by the surgeon to best fit the conduit and the same probe will be used for both ITAs in order to minimize variability and measurement error. The second measurement will be performed after both ITAs have been harvested, are ready for distal division and systemic heparin has been administered. Direct blood flow from each ITA will be measured upon division by measuring the amount of blood filling a calibrated cup over 15 seconds. The ITAs will then be treated with a vasodilator, papaverine, applied topically, in order to eliminate any vasospasm that may occur due to the handling as well as mechanical or thermal trauma to the vessel. The third flow measurement will be performed after a 5–10 minute treatment with papaverine (equal duration of treatment for both ITAs) using the direct flow technique described above. The patient will then undergo coronary artery bypass grafting and the final flow measurement will be performed after all anastomoses have been completed, using the Doppler flow probe.

The amount of flow within a vessel is a function of the systemic blood pressure and the resistance offered by the vessel. In addition, errors in measurement exist with all measuring devices including the transonic Doppler flow probe. Post-anastomotic Doppler flows are further dependent on the size of the outflow vessel, the degree of distal disease and the size and vasoreactive characteristics of the distal myocardial bed that is supplied. Recognizing these potential sources of inaccuracies, pre-anastomotic direct flow (before papaverine treatment) measured from both ITAs at approximately the same time will be used as the primary outcome measure.

### Chest wall pain and dysesthesia

Assessments of chest wall pain and dysesthesia will be performed by a blinded observer. A questionnaire, designed to assess differences in pain between the left and right sides, will be administered to patients, and the degree of pain will be quantified using a 100 mm Visual Analog Scale (VAS), which has been used in numerous disciplines to objectively quantify pain. Patients will also be questioned about the quality and the impact of post-operative pain on their daily activities as well as the need for analgesic medication. However, only the VAS quantification of pain will be side-specific.

**Figure 2:**
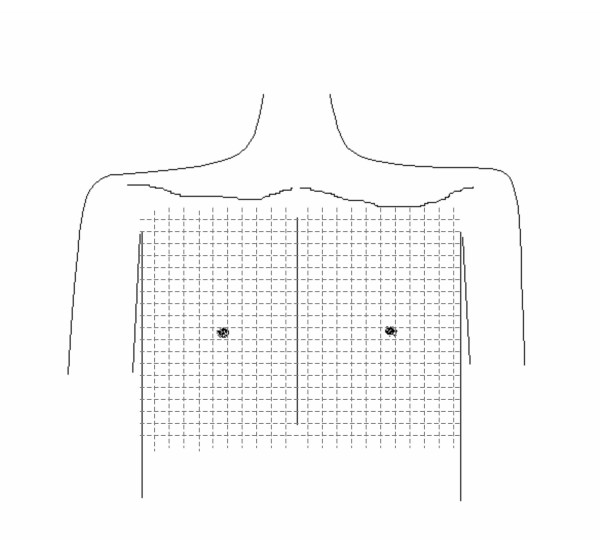
This diagram depicts the anterior chest wall grid to be used for mapping of sensory deficits. Separate grids will be used for the mapping of defects in light touch, pin-prick, and temperature by a blinded observer and areas of anesthesia and hyperesthesia will be mapped using different symbols.

There is a paucity of data on the optimal methodology for assessing and quantifying post-operative dysesthesia of the chest wall following ITA harvest. In consultation with a pain specialist, we have developed a methodology for the detailed assessment of sensory deficits of the anterior chest wall. A sensory examination will be conducted by a blinded observer employing three different sensory modalities. Deficits in light touch, pinprick sensation, and temperature will be mapped out on a grid (Figure 2) by systematically testing the entire anterior chest wall from the clavicle, superiorly, to the inferior edge of the midline skin incision. These assessments of pain and dysesthesia will be conducted upon discharge (4 – 6 days post-operatively), at 4 weeks, and at 3 months post-operatively.

### Sternal ischemia

A subgroup of patients (n = 15) will be randomly chosen to undergo Single Photon Emission Computed Tomography (SPECT) to assess sternal vascularity. Each study involves complete tomographic imaging of the sternum pre and post operatively (between post-operative days 3 and 5). The scan involves the use of ^99m^Tc labeled methylene diphosphonate, and the results of SPECT imaging will be analyzed as previously using a previously described protocol [6,17], adapted to a paired side to side comparison. The results will be reported as counts/pixel.

### Safety endpoints and other data collection

In addition to the outcome measures described, several prospectively defined safety endpoints will be monitored. These include ITA harvest time, gross ITA injury, sternal fractures, chest tube drainage, need for re-operation for bleeding, as well as superficial or deep sternal wound infections. Information on baseline characteristics, intra-operative variables and post-operative outcomes will also be collected prospectively.

### Statistical analysis and sample size calculations

The patient characteristics will be described using means ± SEM or medians (interquartile range) for normally distributed and skewed data as appropriate. Analysis of all endpoints will be conducted using paired analysis. Continuous variables will be analyzed using 3-way ANOVA, adjusting for stratification and time period variables. Differences in conduit flow (ml/min), length (cm), sternal perfusion (# pixel counts/cm^2^), chest wall pain (VAS score), and area of chest wall dysesthesia (cm^2^) will be analyzed using these methods. In addition, chest wall pain and chest wall dysesthesia will also be dichotomized and analyzed as binary endpoints using McNemar's test. All statistical analyses will be conducted using SAS version 9.1 (SAS Institute, Cary, NC, USA).

For the purpose of sample size calculations, change in ITA flow has been chosen as the primary outcome measure. The reason for this choice is that a) this is a clinically important early endpoint affected by harvesting technique, b) there is some preliminary data available from observational studies evaluating ITA flow, and c) there is a lack of preliminary data regarding other endpoints like chest wall pain and dysesthesia to use for a sample size calculation.

The sample size for this study will be 45 patients i.e. 45 pairs of ITAs. Using direct ITA flow at the second measurement, prior to papaverine application, this study will be powered to detect an absolute difference as low as 8 ml/min between the skeletonized and non-skeletonized ITA flows (30% increase in flow) with the assumption of a mean flow of 26.4 ml/min in the non-skeletonized group[14], at a two-sided alpha level of 0.05 and 90% power. Since the estimate for the standard deviation of the difference between paired samples is not known, the standard deviation between unpaired samples of 16.1 was used to calculate sample size[14]. Since the standard deviation of the difference between paired samples is expected to be less that this value, this likely overestimates the required sample size. There is no anticipated loss to follow-up with respect to the intra-operative ITA flow endpoint.

## Discussion

Although the routine use of the internal thoracic arteries is well established among cardiac surgeons, there is considerable debate regarding the optimal harvesting technique for this conduit. In this paper, we describe the rationale and study protocol, utilizing a paired study design for the evaluation of skeletonized versus non-skeletonized harvest of the internal thoracic artery. The primary outcome measure for this study is intra-operative ITA flow. Additional outcome measures include ITA length, sternal perfusion, and post-operative pain and dysesthesias. The strengths of this study include the assessment of multiple clinically relevant endpoints using an efficient study design with within-patient comparisons.

### Limitations of ITA use

The use of ITAs as bypass conduits has been demonstrated to improve clinical outcomes following coronary artery bypass surgery. However, there are several potential reasons that limit the utilization of the ITA. First, the pre-disposition of the ITAs to post-operative vasospasm and hypoperfusion can potentially be amplified in the presence of vasoactive medications and therefore, there is some reluctance to using these conduits in patients undergoing emergency surgery as well as those with poor left ventricular function. Catastrophic consequences of ITA hypoperfusion, including death and cardiac transplantation, have been reported[4]. Second, there is a concern that ITA harvest may compromise sternal healing. Clinical studies have established the association between bilateral ITA harvest and sternal wound infection, particularly in diabetic patients[18]. Post-operative sternal infection and mediastinitis have severe consequences including increased mortality, prolonged intensive care unit and in-hospital stay, and increased economic burden[7]. The third factor relates to the length of usable conduit available. The distal, post-bifurcation ITA is more prone to vasospasm and its use has been linked to early graft occlusion[5]. Although sequential arterial grafting is a strategy that successfully reduces the amount of conduit needed, one of the limitations to multi-vessel arterial grafting is the length of available conduit[19]. Finally, due to the close proximity of the ITA to cutaneous nerves of the chest wall, its harvest by traditional techniques has been associated with significant persistent pain and sensory chest wall changes, also termed the 'internal thoracic artery syndrome'[9]. Various studies have reproduced the finding of increased post-operative pain in patients undergoing ITA harvest versus sternotomy alone[8,10].

### Proposed benefits of skeletonization

The traditional technique for ITA harvest involves a wide dissection of tissue around the artery (approximately 1 to 2 cm) so as to include the internal thoracic vein and surrounding muscle, fat, pleura and connective tissue as part of the ITA pedicle. On the other hand, skeletonization, which involves the harvest of only the artery, has been reported to result in increased ITA length[12], larger luminal diameter[14], and higher ITA flows[20,21]. These assertions are supported primarily by non-randomized, observational studies, which are prone to bias as well as affected by unmeasured confounders that differ between patients e.g. patient size, gender, use of vasoactive medication, and hemodynamic status. One clinical study has also suggested increased preservation of the sternal blood supply with single skeletonized ITA harvest[17]. Whether this reduction in sternal ischemia leads to a decreased rate of sternal wound infection is unknown. There is a suggestion from observational studies that the incidence of sternal complications in patients undergoing bilateral skeletonized ITA harvest is lower than the currently reported rates for conventional bilateral ITA harvesting[15,22]. Only one observational study to date has examined early post-operative pain with the finding of increased pain in the non-skeletonized group[16]. Thus, there is a lack of conclusive data on the proposed benefits of ITA skeletonization.

### Paired, within-patient studies in surgery

The vast majority of randomized clinical trials utilize a parallel study design to assess treatment differences between groups. The paired study design is efficient because it eliminates the effects of between-patient variability and therefore, significantly reduces the number of patients required to detect a difference in the primary endpoint. This design shares many features of cross-over studies, which are often used to assess effects of medications (e.g. blood pressure lowering medications) that are relatively short acting and being used to treat a disease that is relatively stable over time (e.g. hypertension). In cross-over studies, all patients receive both treatments, and the order in which treatments are administered is randomly assigned. The major concerns with this study design are a) carry over effects i.e. persistence of the effects of the first treatment while the second treatment is being administered, and b) changes in the disease state over the treatment periods. The addition of a washout period between treatments, when no treatment is administered, helps to allay some of this concern[23]. In addition to the two period cross-over design, more complicated cross-over designs that can evaluate multiple treatments have also been proposed[24].

Evaluation of surgical interventions is rarely amenable to a cross-over design. However, occasionally the presence of anatomic symmetry within a patient allows for a within-patient comparison. One of the earliest applications of this type of design in a surgical setting was the use of laser photocoagulation in the eyes of diabetic patients using the untreated eye as controls[25]. Our study design shares a number of features of a cross-over study in that each patient receives both treatments, the order in which treatments are administered is randomized, and the outcomes are assessed according to treatment assignment in a side-specific manner. Although it provides an efficient study design, an important implicit assumption of this design is that the organs that receive the intervention are the same i.e. in this case, that the left and right ITAs are the same, both at the pre-treatment stage and with respect to their response to treatment. Although this assumption seems intuitively reasonable, there are certain anatomic differences between the origins of the left and right ITAs that may question this assumption and this is a limitation of this study design. To address this issue, the randomization scheme will stratify according to side (left versus right) to ensure equivalent numbers in both groups.

Another limitation is the inability to assess the effects of skeletonization on sternal wound infections and other endpoints that are not side-specific. Nevertheless, this design allows for the control of various systemic factors that are identical between the two ITAs. These factors include patient size, the presence of diabetes, hypertension, and other cardiovascular risk factors, the systemic consequences of surgical trauma, systemic endogenous release or exogenous administration of vasoactive substances, and particularly important in our case, the subjective pain experience of each individual in response to surgery. A unique feature of this type of paired study is that the treatments are administered at roughly the same time and therefore, period effects are not expected to play a major role in confounding treatment related differences.

### Summary

Skeletonization of the internal thoracic artery is a new surgical technique that can potentially improve the quality of the ITA as a bypass conduit as well as reduce post-operative morbidity. Although advantages of this technique have been suggested in observational studies, definitive data supporting its benefits is lacking. As such, there is significant variation between surgeons in the harvesting technique employed. As cardiac surgeons strive to enhance the efficacy and minimize the morbidity associated with surgical interventions, this study will provide a valid evaluation of clinically relevant effects of internal thoracic artery skeletonization in improving outcomes following coronary artery bypass surgery.

## Competing interests

The author(s) declare that they have no competing interests.

## Authors' contributions

MB, HJN, and FDR were responsible for the design of the study. BKL and FDR will be performing the study interventions.
